# The Novel Positive Allosteric Modulator of the GABA_B_ Receptor, KK-92A, Suppresses Alcohol Self-Administration and Cue-Induced Reinstatement of Alcohol Seeking in Rats

**DOI:** 10.3389/fcell.2021.727576

**Published:** 2021-10-28

**Authors:** Paola Maccioni, Katarzyna Kaczanowska, Harshani Lawrence, Sang Yun, Jessica Bratzu, Gian Luigi Gessa, Patricia McDonald, Giancarlo Colombo

**Affiliations:** ^1^Neuroscience Institute, Section of Cagliari, National Research Council of Italy, Monserrato, Italy; ^2^Department of Chemistry, The Scripps Research Institute, La Jolla, CA, United States; ^3^Chemical Biology Core, Moffitt Cancer Center, Tampa, FL, United States; ^4^Department of Cancer Physiology, Moffitt Cancer Center, Tampa, FL, United States

**Keywords:** KK-92A, positive allosteric modulator, GABA_B_ receptor, alcohol self-administration, cue-induced reinstatement of alcohol seeking, rats

## Abstract

Positive allosteric modulators (PAMs) of the GABA_B_ receptor (GABA_B_ PAMs) are of interest in the addiction field due to their ability to suppress several behaviors motivated by drugs of abuse. KK-92A is a novel GABA_B_ PAM found to attenuate intravenous self-administration of nicotine and reinstatement of nicotine seeking in rats. This present study was aimed at extending to alcohol the anti-addictive properties of KK-92A. To this end, Sardinian alcohol-preferring rats were trained to lever-respond for oral alcohol (15% v/v) or sucrose (0.7% w/v) under the fixed ratio (FR) 5 (FR5) schedule of reinforcement. Once lever-responding behavior had stabilized, rats were exposed to tests with acutely administered KK-92A under FR5 and progressive ratio schedules of reinforcement and cue-induced reinstatement of previously extinguished alcohol seeking. KK-92A effect on spontaneous locomotor activity was also evaluated. Treatment with 10 and 20 mg/kg KK-92A suppressed lever-responding for alcohol, amount of self-administered alcohol, and breakpoint for alcohol. Treatment with 20 mg/kg KK-92A reduced sucrose self-administration. Combination of *per se* ineffective doses of KK-92A (2.5 mg/kg) and the GABA_B_ receptor agonist, baclofen (1 mg/kg), reduced alcohol self-administration. Treatment with 5, 10, and 20 mg/kg KK-92A suppressed reinstatement of alcohol seeking. Only treatment with 80 mg/kg KK-92A affected spontaneous locomotor activity. These results demonstrate the ability of KK-92A to inhibit alcohol-motivated behaviors in rodents and confirm that these effects are common to the entire class of GABA_B_ PAMs. The remarkable efficacy of KK-92A is discussed in terms of its ago-allosteric properties.

## Introduction

Positive allosteric modulation of the GABA_B_ receptor has emerged as an important molecular mechanism to effectively control several alcohol-motivated behaviors. Accordingly, all positive allosteric modulators (PAMs) of the GABA_B_ receptor (GABA_B_ PAMs) tested to date (namely: CGP7930, GS39783, BHF177, *rac*-BHFF, ADX71441, COR659, CMPPE, ORM-27669, and ASP8062) have invariably been reported to reduce excessive alcohol drinking (Orrù et al., 2005; Loi et al., 2013; Hwa et al., 2014; Ferlenghi et al., 2020), binge-like drinking (Hwa et al., 2014; Linsenbardt and Boehm, 2014; Colombo et al., 2015; de Miguel et al., 2019), relapse-like drinking (Vengeliene et al., 2018), operant oral alcohol self-administration (Liang et al., 2006; Maccioni et al., 2007, 2008b, 2009, 2010, 2012, 2015, 2017, 2018, 2019a, b; Augier et al., 2017; Lorrai et al., 2019; Ferlenghi et al., 2020; Haile et al., 2021), cue- and stress-induced reinstatement of alcohol seeking (Augier et al., 2017; Vengeliene et al., 2018; Maccioni et al., 2019a, b), alcohol-induced hyperlocomotion (Kruse et al., 2012), and alcohol-induced conditioned place preference (de Miguel et al., 2019) in rats and mice (for review, see Maccioni and Colombo, 2019; Holtyn and Weerts, 2020).

The pharmacological profile of GABA_B_ PAMs possess numerous advantages, particularly when compared to that of the orthosteric agonist of the GABA_B_ receptor, baclofen. Focusing on alcohol-motivated behaviors, the reducing effects of GABA_B_ PAMs occurred at doses largely lower than those inducing sedation and muscle relaxation (Maccioni et al., 2010, 2017; Linsenbardt and Boehm, 2014; Vengeliene et al., 2018; de Miguel et al., 2019) and devoid of any effect on natural rewards (e.g., water, regular or palatable food) (Orrù et al., 2005; Maccioni et al., 2007, 2008b, 2009, 2010, 2012, 2015, 2019b; Loi et al., 2013; Hwa et al., 2014; Colombo et al., 2015; see however Augier et al., 2017). Additionally, no tolerance developed on continuing treatment (Loi et al., 2013; Maccioni et al., 2015, 2019a; Vengeliene et al., 2018). These favorable features (with undoubted translational value) likely reside in the use-dependent mechanism of action of GABA_B_ PAMs. At variance with baclofen that stimulates each GABA_B_ receptor it encounters, GABA_B_ PAMs only potentiate the receptor activation induced by endogenous GABA, thus limiting their action when and where endogenous GABA is released (see Urwyler, 2011, 2016), resulting *in vivo* in a larger separation between the expected pharmacological effects and putative off-target side-effects. Additionally, the absence of persistent receptor activation (typical, on the other hand, of receptor agonists) results in a low propensity to induce receptor desensitization, explaining why repeated treatment with GABA_B_ PAMs is associated with limited development of tolerance (see Urwyler, 2011, 2016). Together, these data indicate GABA_B_ PAMs as active molecules having an improved therapeutic potential over baclofen.

KK-92A [(4-(cycloheptylamino)-5-(4-(trifluoromethyl)phen yl)pyrimidin-2-yl)methanol] is the final product of a recent project of medicinal chemistry and pharmacology aimed at identifying novel GABA_B_ PAMs starting from the chemical structure of the GABA_B_ PAM, BHF177 (Li et al., 2017). Among the approximately 100 analogs that had been synthesized, KK-92A was identified as the preferred compound because of its (i) high potency and selectivity as a GABA_B_ PAM in multiple *in vitro* cell-based assays, (ii) high bioavailability in the brain, and (iii) remarkable *in vivo* efficacy (specifically, the selective reducing effect on intravenous self-administration of nicotine and cue-induced reinstatement of nicotine seeking in rats) (Li et al., 2017). The in-depth investigation of its *in vitro* pharmacological profile (Li et al., 2017) makes KK-92A one of the best characterized GABA_B_ PAMs available to date and a powerful tool for further investigations of their *in vivo* actions and therapeutic potential.

Accordingly, the present study was designed to investigate whether the reducing effects of GABA_B_ PAMs on alcohol-motivated behaviors are shared by KK-92A. To this end, acutely administered KK-92A was tested in three different, validated experimental procedures of alcohol seeking and drinking: operant oral alcohol self-administration under the fixed ratio (FR) (Experiment 1A) and progressive ratio (PR) (Experiment 2) schedules of reinforcement, that provide a measure of the reinforcing and motivational properties of alcohol, respectively (see Markou et al., 1993), and cue-induced reinstatement of alcohol seeking, that models human loss of control over alcohol and relapse into heavy alcohol drinking (see Martin-Fardon and Weiss, 2013) (Experiment 3). Selectivity of KK-92A effect on alcohol self-administration was evaluated testing acutely administered KK-92A on sucrose self-administration under the FR schedule of reinforcement (Experiment 1B). The present study also included investigation of the effect of acute treatment with the combination of *per se* ineffective doses of KK-92A and baclofen on alcohol self-administration under the FR schedule of reinforcement (Experiment 1C), with the intent of assessing whether treatment with KK-92A potentiated the reducing effect of baclofen on the reinforcing properties of alcohol. In an attempt to exclude the possibility that the effects of KK-92A on the above alcohol- and sucrose-motivated behaviors were due to sedative and motor-incoordinating effects of KK-92A (a not unlikely event when testing a drug that targets GABA neurotransmission), Experiment 4 evaluated the effect of acute treatment with KK-92A on spontaneous locomotor activity. The effect of acute treatment with KK-92A on blood alcohol levels (BALs) was also assessed (Experiment 5).

All experiments were conducted using the Sardinian alcohol-preferring (sP) rats, one of the few rat lines selectively bred for high alcohol preference and consumption (see Colombo et al., 2006; Bell et al., 2012). sP rats meet all the fundamental requirements posed when defining an animal model of alcohol use disorder (AUD) (see Colombo et al., 2006; Bell et al., 2012). Notably, in relation to the aims of the present study, several previous studies indicated that alcohol self-administration and cue-induced reinstatement of alcohol seeking in sP rats were highly sensitive to positive allosteric modulation of the GABA_B_ receptor (Maccioni et al., 2007, 2008b, 2009, 2010, 2012, 2015, 2017, 2018, 2019a, b; Lorrai et al., 2019; Ferlenghi et al., 2020).

## Materials and Methods

The experimental procedures employed in the present study fully complied with European Directive no. 2010/63/EU and subsequent Italian Legislative Decree no. 26, March 4, 2014, on the “Protection of animals used for scientific purposes.”

### Animals

Female sP rats (bred in our laboratory at Neuroscience Institute, Section of Cagliari, National Research Council of Italy, Italy) were used. Rats were 50-days-old at the start of each experiment, from 110th to 112th generation, and alcohol-naive at the start of each experiment. Rats were housed three per cage in standard plastic cages with wood chip bedding. The animal facility was under an inverted 12:12-h light-dark cycle (lights on at 7:00 p.m.), at a constant temperature of 22 ± 2°C and relative humidity of approximately 60%. Standard rat chow and tap water were always available in the homecage, except as noted below. Rats were extensively habituated to handling, intraperitoneal injections, and intragastric infusions (the latter limited to rats allocated to Experiment 5).

Female rats were preferred over male rats as their body weight is more stable and much lower than adult male sP rats, resulting in the several practical advantages described elsewhere (Lorrai et al., 2019). Importantly, sensitivity of alcohol self-administration to pharmacological manipulation is highly similar in female and male sP rats: as an example, acute treatment with the GABA_B_ PAM, GS39783, reduced alcohol self-administration under the FR schedule with comparable potency and efficacy in female and male sP rats (Lorrai et al., 2019).

To avoid any possibility of ovarian hormones influencing alcohol and sucrose self-administration (Experiments 1A-C and 2), reinstatement of alcohol seeking (Experiment 3), and alcohol metabolism (Experiment 5), rats were ovariectomized. Ovariectomy was performed when rats were 45 days old and according to the procedure described in detail elsewhere (Lorrai et al., 2019). A recovery period of 5 days following surgery occurred before the start of the alcohol-drinking phase (see below). For reasons of uniformity and consistency among the five experiments, ovariectomy was also performed in rats allocated to Experiment 4 (spontaneous locomotor activity).

Each single experiment used an independent set of rats.

### Drugs

KK-92A was synthesized in gram-scale with >99% purity (as determined by HPLC) in the Chemical Biology Core laboratory at Moffitt Cancer Center, FL, United States, according to the procedure described in detail by Li et al. (2017). The chemical analysis (^1^H and ^13^C NMR, HPLC-MS) of in-house synthesized KK-92A matched the reported data (see Supplementary Material for structure and chemical analysis). KK-92A was dissolved in a mixture containing dimethyl sulfoxide, polysorbate 80, and distilled water (ratio of the mixture components: 5:10:85) for *in vivo* assessment. In all experiments, KK-92A was administered acutely and intraperitoneally (i.p.; injection volume: 2 ml/kg) 30 min before (a) start of self-administration (Experiments 1A-C and 2), reinstatement (Experiment 3), and locomotor-activity (Experiment 4) sessions and (b) alcohol administration (Experiment 5). In Experiments 1A, 1B, 2, 3, and 5, KK-92A was tested at doses of 0, 5, 10, and 20 mg/kg; this dose range was chosen to be identical to that previously tested on nicotine self-administration and reinstatement of nicotine seeking in Wistar rats (Li et al., 2017). In Experiment 1C, KK-92A was tested at the doses of 0 and 2.5 mg/kg; the latter was chosen on the basis of preliminary data suggesting that it was totally ineffective, when given alone, on alcohol self-administration in sP rats (this laboratory, unpublished results). In Experiment 4, KK-92A was tested at the doses of 0, 20, 40, and 80 mg/kg; this larger dose range was chosen to identify possible sedative and motor-incoordinating effects.

Baclofen (Novartis, Basel, Switzerland) was dissolved in saline and injected i.p. (injection volume: 2 ml/kg) at the doses of 0 and 1 mg/kg 30 min before the start of the test session of Experiment 1C. Pretreatment time and route of administration were identical to those used in previous studies testing baclofen on alcohol self-administration in sP rats (Maccioni et al.2005, 2008, 2012; 2015). Dosage was selected as being totally ineffective, when given alone, on alcohol self-administration in sP rats (Maccioni et al., 2012, 2015).

### Alcohol or Sucrose Self-Administration and Cue-Induced Reinstatement of Alcohol Seeking

#### Apparatus

Self-administration, extinction responding, and reinstatement sessions were conducted in modular chambers (Med Associates, St. Albans, VT, United States) described in detail elsewhere (e.g., Maccioni et al., 2015). Briefly, each chamber was equipped with two retractable response levers (connected to two syringe pumps located outside the chamber), one dual-cup liquid receptacle, two stimulus lights (mounted above each lever), and one tone generator.

In self-administration sessions, achievement of the response requirement (RR) had the following consequences: activation of alcohol (or sucrose) or water pumps, delivery of 0.1 ml fluid, illumination of the stimulus light for the time period of fluid delivery, and activation of the tone generator.

#### Experimental Procedure

##### Training and maintenance phases of alcohol or sucrose self-administration

In alcohol self-administration experiments, rats were initially exposed to the homecage 2-bottle “alcohol (10% v/v) vs. water” choice regimen with unlimited access for 24 h/day over 10 consecutive days, according to the procedure described in detail elsewhere (e.g., Maccioni et al., 2015). Subsequently, rats were introduced into the operant chambers and trained to lever-respond for alcohol. Self-administration sessions lasted 30 min (with the sole exception of the very first session, that lasted 120 min) and were conducted 5 days per week. Rats were water-deprived exclusively during the 12 h prior to the first session in the operant chamber. Rats were initially exposed to an FR1 schedule of reinforcement for 10% alcohol (v/v) for four sessions. FR was then progressively increased to FR5 over four sessions. In sessions 9 and 10, the alcohol solution was presented at a final concentration of 15% (v/v). Rats were then exposed to four sessions during which the water lever alone or alcohol lever alone was available every other day; water and alcohol were available on FR1 and FR5, respectively. From then onward, both levers were concomitantly available (maintenance phase) for a total of 20 sessions conducted with FR5 and FR1 on the alcohol and water lever, respectively. On completion of the maintenance phase, rats displaying the most stable responding behavior were selected for use in Experiments 1A, 1C, 2, and 3.

In the sucrose self-administration experiment, rats were trained to lever-respond for a sucrose solution. Self-administration sessions lasted 30 min (with the sole exception of the very first session, that lasted 120 min) and were conducted 5 days per week. Rats were water-deprived exclusively during the 12 h prior to the first session in the operant chamber. Rats were initially exposed to an FR1 schedule of reinforcement for 2% (w/v) sucrose solution (in water) for four sessions. FR was then progressively increased to FR5 over four sessions. Sucrose concentration was reduced to 0.7% (w/v) over six sessions. This sucrose concentration was selected on the basis of previous results (e.g., Maccioni et al., 2010) in order to establish a lever-responding behavior comparable to that usually performed by sP rats to obtain 15% alcohol under FR5. Rats were then exposed to four sessions during which the water lever alone or the sucrose lever alone was available every other day; water and sucrose were available on FR1 and FR5, respectively. From then onward, both levers were concomitantly available (maintenance phase) for a total of 20 sessions conducted with FR5 and FR1 on the sucrose and water lever, respectively. On completion of the maintenance phase, the rats displaying the most stable responding behavior were selected for use in Experiment 1B.

##### Testing under the fixed ratio schedule

Experiment 1A evaluated the effect of acute treatment with different doses of KK-92A on alcohol self-administration under the FR5 (alcohol) and FR1 (water) schedule of reinforcement. This experiment employed a total of *n* = 48 rats (selected as described above from an original set of *n* = 56), divided into four groups of *n* = 12 matched for the number of responses on the alcohol lever over the last three sessions of the maintenance phase.

Experiment 1B evaluated the effect of acute treatment with different doses of KK-92A on sucrose self-administration under the FR5 (sucrose) and FR1 (water) schedule of reinforcement. This experiment employed a total of *n* = 44 rats (from an original set of *n* = 50), divided into four groups of *n* = 11 matched for the number of responses on the sucrose lever over the last three sessions of the maintenance phase.

Experiment 1C evaluated the effect of the combination of *per se* ineffective doses of KK-92A and baclofen on alcohol self-administration under the FR5 (alcohol) and FR1 (water) schedule of reinforcement. This experiment employed a total of *n* = 48 rats (from an original set of *n* = 56), divided into four groups of *n* = 12 matched for the number of responses on the alcohol lever over the last three sessions of the maintenance phase. The following four treatment combinations were tested: 0 mg/kg KK-92A + 0 mg/kg baclofen; 0 mg/kg KK-92A + 1 mg/kg baclofen; 2.5 mg/kg KK-92A + 0 mg/kg baclofen; 2.5 mg/kg KK-92A + 1 mg/kg baclofen.

In all three experiments, the test session occurred the day after completion of the maintenance phase, lasted 30 min, and was identical to those of the maintenance phase [FR5 and FR1 on the alcohol (or sucrose) and water lever, respectively].

Measured variables were: (a) number of responses on each lever; (b) amount of self-administered alcohol (expressed in g/kg pure alcohol) or sucrose solution (expressed in ml/kg), estimated from the number of earned reinforcers assuming that each reinforcer was entirely consumed. In Experiment 1A, latency (expressed in s) to the first alcohol reinforcer was also measured; rats that completely avoided responding on the lever were assigned the value 1,800 s (i.e., the entire length of the test session). Data on number of responses on each lever and amount of self-administered alcohol (or sucrose solution) were statistically evaluated by 1-way ANOVA with repeated measures, followed by Tukey’s test for *post hoc* comparisons. Data on latency to the first alcohol reinforcer were statistically evaluated by Kruskal-Wallis test, followed by Dunn’s for *post hoc* comparison.

##### Testing under the progressive ratio schedule

Experiment 2 evaluated the effect of acute treatment with different doses of KK-92A on alcohol self-administration under the PR schedule of reinforcement. This experiment employed a total of *n* = 48 rats (from an original set of *n* = 56), divided into four groups of *n* = 12 matched for the number of responses on the alcohol lever over the last three sessions of the maintenance phase. The test session occurred the day after completion of the maintenance phase and lasted 60 min. In the test session, RR on the alcohol lever was increased progressively over the session according to a procedure slightly adapted from that described by Richardson and Roberts (1996); namely, RR was increased as follows: 5, 9, 12, 15, 20, 25, 32, 40, 50, 62, 77, 95, 118, 145, 178, 219, etc. The water lever was inactive.

Measured variables were: (a) number of responses on each lever; (b) breakpoint for alcohol, defined as the lowest RR not achieved by the rat; (c) latency (expressed in s) to the first reinforcer (rats that completely avoided responding on the lever were assigned the value 3,600 s, i.e., the entire length of the test session). Data from each variable were statistically evaluated by 1-way ANOVA with repeated measures, followed by Tukey’s test for *post hoc* comparisons.

##### Testing under the reinstatement of alcohol-seeking protocol

Experiment 3 evaluated the effect of acute treatment with different doses of KK-92A on cue-induced reinstatement of alcohol seeking. To this end, immediately after completion of the maintenance phase, rats underwent an extinction-responding phase made up of consecutive (no weekend interruption) daily sessions (lasting 60 min) characterized by unavailability of alcohol and water; specifically, syringe pumps, stimulus lights, and tone generator were off, and lever-responding was unreinforced. An extinction criterion was set at ≤12 responses on the alcohol lever per session for two consecutive sessions (Maccioni et al., 2019b).

This experiment employed a total of *n* = 30 rats (from an original set of *n* = 40), divided into four groups of *n* = 7–8 matched for the number of responses on the alcohol lever over the first three sessions of the extinction-responding phase. The day after achievement of the extinction criterion, each rat was exposed to a single 60-min reinstatement (test) session, during which a stimulus complex—previously associated to availability of alcohol—was presented for 10 times within 20 s. This stimulus complex was composed of tone, turning on of the stimulus lights, and availability, every other time, of 0.1 ml alcohol (15% v/v) in the liquid receptacle (for a total number of 5 presentations). Immediately after the last presentation of the stimulus complex, both levers were inserted inside the chamber and lever-responding (still unreinforced) was recorded.

The measured variable was the number of responses on alcohol lever during the reinstatement session. Data were statistically evaluated by 2-way [phase (extinction/reinstatement); treatment (KK-92A dose)] ANOVA with repeated measures on the factor “phase,” followed by Bonferroni’s test for *post hoc* comparisons. An additional analysis evaluated the number of sessions of the extinction responding phase needed to achieve the extinction criterion; these data were analyzed by 1-way ANOVA and log-rank (Mantel-Cox) test.

### Locomotor Activity

#### Apparatus

Locomotor activity (ambulation) was measured in Plexiglass test cages [480 × 480 × 400 (h) mm] by a computer-operated, photocell-equipped apparatus (Motil, TSE, Bad Homburg, Germany). Photocells were 40-mm spaced. Test cages were located in a sound-proof, dimly lit room adjacent to the housing room.

#### Experimental Procedure

Experiment 4 evaluated the effect of acute treatment with different doses of KK-92A on spontaneous locomotor activity. Rats were initially exposed to the homecage 2-bottle “alcohol (10% v/v) vs. water” choice regimen with unlimited access for 24 h/day throughout 10 consecutive days. Subsequently, rats were trained to lever-respond for alcohol using the same procedure described above. Consequently, the “alcohol” history of these rats was identical to that of the rats used in Experiments 1A, 1C, 2, 3, and 5.

This experiment employed a total of *n* = 39 rats, divided into four groups of *n* = 9–10 matched for body weight and number of responses on the alcohol lever over the last three sessions of the maintenance phase. The locomotor-activity test was conducted the day after completion of the maintenance phase and lasted 30 min. Rats were unfamiliar to the motility cage, in order to provide relatively high baseline levels of spontaneous locomotor activity (i.e., a desirable condition to amplify the possible suppressing effect of the tested drug) (see Kelley, 1993).

The measured variable was the number of motility counts (photocell breaks), recorded automatically by the apparatus. Data were divided into six 5-min time intervals and statistically analyzed by a 2-way (KK-92A dose; time) ANOVA with repeated measures on the factor “time,” followed by Tukey’s test for *post hoc* comparisons. The total (cumulated) number of motility counts over the entire session was statistically evaluated by 1-way ANOVA, followed by Tukey’s test for *post hoc* comparisons.

### Blood Alcohol Levels

#### Apparatus

Blood samples were analyzed by means of an enzymatic system [GL5 Analyzer (Analox Instruments, London, United Kingdom)] based on measurement of oxygen consumption in the alcohol-acetaldehyde reaction.

#### Experimental Procedure

Experiment 5 evaluated the effect of acute treatment with different doses of KK-92A on BALs. Rats were initially exposed to the homecage 2-bottle “alcohol (10% v/v) vs. water” choice regimen with unlimited access for 24 h/day throughout 10 consecutive days. Subsequently, rats were trained to lever-respond for alcohol using the same procedure described above. Consequently, the “alcohol” history of these rats was identical to that of the rats used in Experiments 1A, 1C, 2, 3, and 4.

This experiment employed a total of *n* = 40 rats, divided into four groups on *n* = 10 matched for body weight and number of responses on the alcohol lever over the last three sessions of the maintenance phase. The experiment was conducted the day after completion of the maintenance phase. Food pellets were removed 4 h before the experiment, to ensure that rats had empty stomachs at the time of alcohol infusion. Thirty min after treatment with KK-92A, rats were treated intragastrically with 1 g/kg alcohol (15% v/v). Blood samples (50 μL) were collected from the tip of the tail of each rat at 30, 60, 120, and 240 min after alcohol administration.

The measured variable was BALs (expressed in mg%). Data on BAL time-course were statistically evaluated by 2-way (KK-92A dose; time) ANOVA with repeated measures on the factor “time,” followed by Tukey’s test for *post hoc* comparisons. Data on the area under the curve of BAL time-course [expressed as (h^∗^μg/ml)] were statistically evaluated by 1-way ANOVA, followed by Tukey’s test for *post hoc* comparisons.

## Results

### Experiment 1A: Testing KK-92A on Alcohol Self-Administration Under the FR5 Schedule

**FIGURE 1:**
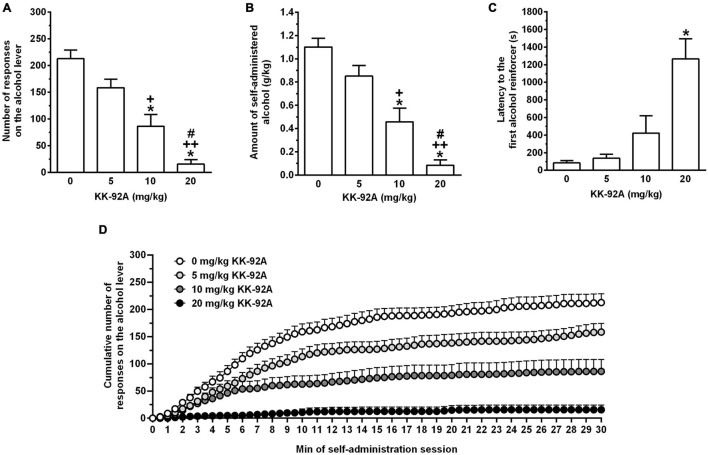
Effect of acute treatment with the positive allosteric modulator of the GABA_B_ receptor, KK-92A, on number of lever-responses for alcohol **(A)**, amount of self-administered alcohol **(B)**, latency to the first reinforcer on the alcohol lever **(C)**, and cumulative response patterns of alcohol self-administration **(D)** in female Sardinian alcohol-preferring rats. Rats were initially trained to lever-respond for oral alcohol (15% v/v in water) [Fixed Ratio (FR) 5 (FR5)] and water (FR1) in daily 30-min self-administration sessions. Once lever-responding had stabilized, rats were tested with KK-92A under the same FR schedule of reinforcement. KK-92A was administered intraperitoneally 30 min before the start of the self-administration session. In panel **(D)**, the self-administration session was divided into 30 intervals of 1 min each. Each bar or point is the mean ± SEM of *n* = 12 rats. *P < 0.0001 in comparison to the rat group treated with 0 mg/kg KK-92A [Tukey’s test in panels **(A,B)**; Dunn’s test in panel **(C)**]; ^+^*P* < 0.05 and ^++^*P* < 0.0001 in comparison to the rat group treated with 5 mg/kg KK-92A (Tukey’s test); ^#^*P* < 0.05 in comparison to the rat group treated with 10 mg/kg KK-92A (Tukey’s test).

Acute treatment with KK-92A suppressed, in a dose-related manner, the number of lever-responses for alcohol [*F*(3, 44) = 27.39, *P* < 0.0001] in female sP rats exposed to the FR5 schedule of reinforcement (Figure 1A). *Post hoc* test indicated that statistical significance was reached by treatment with 10 (*P* < 0.0001) and 20 (*P* < 0.0001) mg/kg KK-92A. The magnitude of the suppressing effect of 10 and 20 mg/kg KK-92A on number of lever-responses for alcohol averaged approximately 60 and 95%, respectively. Suppression in number of lever-responses for alcohol resulted in a proportional decrease in the amount of self-administered alcohol [*F*(3, 44) = 26.42, *P* < 0.0001] (Figure 1B). At *post hoc* test, statistical significance was reached by treatment with 10 (*P* < 0.0001) and 20 (*P* < 0.0001) mg/kg KK-92A. Acute treatment with KK-92A increased latency to the first alcohol reinforcer [*F*(3, 44) = 17.85, *P* < 0.0005] (Figure 1C). *Post hoc* test indicated that statistical significance was reached only by treatment with 20 mg/kg KK-92A (*P* < 0.0005). After treatment with 20 mg/kg KK-92A, latency to the first alcohol reinforcer was increased by approximately 15 times.

Lever-responding for water was negligible (averaging < 3 per session in all rat groups) and not altered by drug treatment (data not shown).

### Experiment 1B: Testing KK-92A on Sucrose Self-Administration Under the FR5 Schedule

**FIGURE 2:**
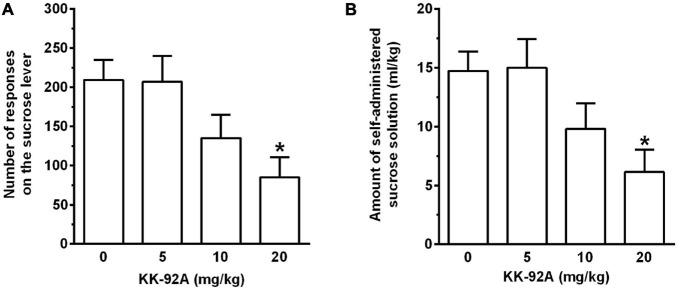
Effect of acute treatment with the positive allosteric modulator of the GABA_B_ receptor, KK-92A, on number of lever-responses for sucrose solution **(A)** and amount of self-administered sucrose solution **(B)** in female Sardinian alcohol-preferring rats. Rats were initially trained to lever-respond for sucrose solution (0.7% w/v in water) [Fixed Ratio (FR) 5 (FR5)] and water (FR1) in daily 30-min self-administration sessions. Once lever-responding had stabilized, rats were tested with KK-92A under the same FR schedule of reinforcement. KK-92A was administered intraperitoneally 30 min before the start of the self-administration session. Each bar is the mean ± SEM of *n* = 11 rats. *P < 0.05 in comparison to the rat group treated with 0 mg/kg KK-92A (Tukey’s test).

Acute treatment with KK-92A reduced, in a dose-related manner, the number of lever-responses for sucrose solution [*F*(3, 40) = 4.44, *P* < 0.01] in female sP rats exposed to the FR5 schedule of reinforcement (Figure 2A). *Post hoc* test indicated that statistical significance was reached only by treatment with 20 mg/kg KK-92A (*P* < 0.05). The magnitude of the suppressing effect of 20 mg/kg KK-92A on number of lever-responses for sucrose solution averaged approximately 60%. Reduction in number of lever-responses for sucrose solution resulted in a proportional decrease in the amount of self-administered sucrose solution [*F*(3, 40) = 4.27, *P* < 0.05] (Figure 2B). At *post hoc* test, statistical significance was reached only by treatment with 20 mg/kg KK-92A (*P* < 0.05).

Lever-responding for water was negligible (averaging < 2 per session in all rat groups) and not altered by treatment with KK-92A (data not shown).

### Experiment 1C: Testing the Combination of KK-92A and Baclofen on Alcohol Self-Administration Under the FR5 Schedule

**FIGURE 3:**
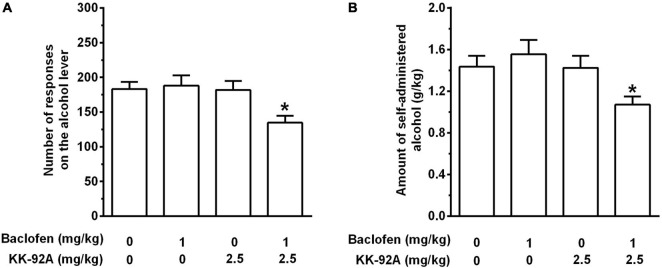
Effect of acute treatment with the combination of *per se* ineffective doses of the positive allosteric modulator of the GABA_B_ receptor, KK-92A, and the GABA_B_ receptor agonist, baclofen, on number of lever-responses for alcohol **(A)** and amount of self-administered alcohol **(B)** in female Sardinian alcohol-preferring rats. Rats were initially trained to lever-respond for oral alcohol (15% v/v in water) [Fixed Ratio (FR) 5 (FR5)] and water (FR1) in daily 30-min self-administration sessions. Once lever-responding had stabilized, rats were tested with all treatment combinations under the same FR schedule of reinforcement. KK-92A and baclofen were administered intraperitoneally 30 min before the start of the self-administration session. Each bar is the mean ± SEM of *n* = 12 rats. *P < 0.05 in comparison to all other rat groups (Tukey’s test).

Acute treatment with the combination of KK-92A and baclofen reduced the number of lever-responses for alcohol [*F*(3, 44) = 4.23, *P* < 0.05] in female sP rats exposed to the FR5 schedule of reinforcement (Figure 3A). Neither KK-92A nor baclofen, when administered alone (or, more precisely, together with the vehicle of the other drug), altered the number of lever-responses for alcohol. Conversely, treatment with the combination of KK-92A and baclofen resulted in an approximately 30% reduction, in comparison to all other three rat groups (*P* < 0.05), in number of lever-responses for alcohol. Reduction in number of lever-responses for alcohol resulted in a proportional decrease in the amount of self-administered alcohol [*F*(3, 44) = 3.56, *P* < 0.05] (Figure 3B). Neither KK-92A nor baclofen, when administered alone, altered the amount of self-administered alcohol. Conversely, treatment with the combination of KK-92A and baclofen resulted in an approximately 25% reduction, in comparison to all other three rat groups (*P* < 0.05), in amount of self-administered alcohol.

Lever-responding for water was negligible (averaging < 2 per session in all rat groups) and not altered by treatment with KK-92A (data not shown).

### Experiment 2: Testing KK-92A on Alcohol Self-Administration Under the Progressive Ratio Schedule

**FIGURE 4:**
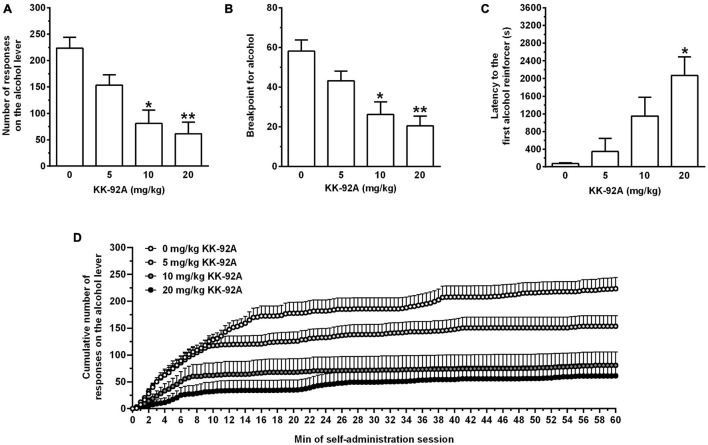
Effect of acute treatment with the positive allosteric modulator of the GABA_B_ receptor, KK-92A, on number of lever-responses for alcohol **(A)**, breakpoint for alcohol **(B)**, latency to the first response on the alcohol lever **(C)**, and cumulative response patterns of alcohol self-administration **(D)** in female Sardinian alcohol-preferring rats. Rats were initially trained to lever-respond for oral alcohol (15% v/v, in water) [Fixed Ratio (FR) 5 (FR5)] and water (FR1) in daily 30-min self-administration sessions. Once lever-responding had stabilized, rats were tested with KK-92A under a progressive ratio schedule of reinforcement, in which the response requirement (RR) was increased progressively over a 60-min session. Breakpoint was defined as the lowest RR not achieved by the rat. KK-92A was administered intraperitoneally 30 min before the start of the self-administration session. In panel **(D)**, the self-administration session was divided into 60 intervals of 1 min each. Each bar or point is the mean ± SEM of *n* = 12 rats. *P < 0.001 and **P < 0.0001 in comparison to the rat group treated with 0 mg/kg KK-92A (Tukey’s test).

Acute treatment with KK-92A reduced, in a dose-related manner, the number of lever-responses for alcohol [*F*(3, 44) = 11.46, *P* < 0.0001] in female sP rats exposed to the PR schedule of reinforcement (Figure 4A). *Post hoc* test indicated that statistical significance was reached by treatment with 10 (*P* < 0.0005) and 20 (*P* < 0.0001) mg/kg KK-92A. The magnitude of the suppressing effect of 10 and 20 mg/kg KK-92A on number of lever-responses for alcohol averaged approximately 65 and 75%, respectively. Acute treatment with KK-92A also reduced, in a dose-related manner, breakpoint for alcohol [*F*(3, 44) = 9.72, *P* < 0.0001] (Figure 4B). *Post hoc* test indicated that statistical significance was reached by treatment with 10 (*P* < 0.001) and 20 (*P* < 0.0001) mg/kg KK-92A. The magnitude of the suppressing effect of 10 and 20 mg/kg KK-92A on breakpoint for alcohol averaged approximately 55 and 65%, respectively. Acute treatment with KK-92A markedly increased latency to the first reinforcer [*F*(3, 44) = 7.29, *P* < 0.0005] (Figure 4C). *Post hoc* test indicated that statistical significance was reached only by treatment with 20 mg/kg KK-92A (*P* < 0.001). After treatment with 20 mg/kg KK-92A, latency to the first alcohol reinforcer was increased by approximately 28 times.

Responding on the inactive lever was modest (averaging < 11 per session in all rat groups) and not altered by treatment with KK-92A (data not shown).

### Experiment 3: Testing KK-92A on Cue-Induced Reinstatement of Alcohol Seeking

**FIGURE 5:**
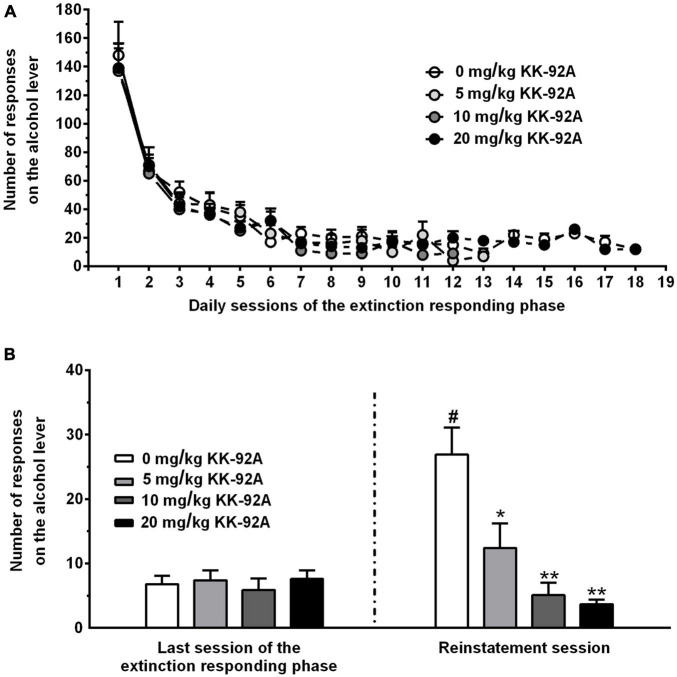
Effect of acute treatment with the positive allosteric modulator of the GABA_B_ receptor, KK-92A, on cue-induced reinstatement of alcohol-seeking behavior in female Sardinian alcohol-preferring rats. Rats were initially trained to lever-respond for oral alcohol (15% v/v, in water) [Fixed Ratio (FR) 5 (FR5)] and water (FR1) in daily 30-min self-administration sessions. Once lever-responding had stabilized, rats were exposed to an extinction responding phase **(A)** during which lever-responding was unreinforced. The reinstatement session occurred once each single rat had achieved the extinction criterion (≤12 responses on the alcohol lever per session for 2 consecutive sessions). In the reinstatement session **(B)**, unreinforced lever-responding was resumed by the repeated presentation of a complex of visual, auditory, gustatory, and olfactory stimuli previously associated with alcohol availability. The reinstatement session lasted 60 min. KK-92A was administered intraperitoneally 30 min before the start of the reinstatement session. In panel **(A)**, each point is the mean ± SEM of an *n* value varying from 7 to 8 in the first extinction-responding sessions to 2 in some of the last extinction-responding sessions (note that in some of these last sessions the sample size of the 20 mg/kg KK-92A-treated rat group was limited to *n* = 1). In panel **(B)**, each bar is the mean ± SEM of *n* = 7–8 rats. ^#^*P* < 0.0001 in comparison to the same rat group in the last session of the extinction-responding phase (Bonferroni’s test); *P < 0.0005 and **P < 0.0001 in comparison to the rat group treated with 0 mg/kg KK-92A in the reinstatement session (Tukey’s test).

Regarding the extinction-responding phase, Log-rank (Mantel-Cox) test indicated that the profile of lever-responding did not differ among the four groups of female sP rats subsequently treated with 0, 5, 10, and 20 mg/kg KK-92A and then exposed to the reinstatement session (χ^2^ = 1.197, *P* > 0.05) (Figure 5A). Additionally, the four rat groups did not differ in number of extinction-responding sessions needed to achieve the extinction criterion [10.6 ± 1.3, 9.7 ± 0.7, 8.9 ± 0.5, and 9.3 ± 2.1 (mean ± SEM) in rats subsequently treated with 0, 5, 10, and 20 mg/kg KK-92A, respectively; *F*(3, 26) = 0.78, *P* > 0.05].

Regarding the reinstatement session, ANOVA indicated significant effects of presentation of the alcohol-associated stimulus complex [*F*(1, 26) = 8.33, *P* < 0.01] and treatment with KK-92A [*F*(3, 26) = 11.14, *P* < 0.0001], and a significant interaction [*F*(3, 26) = 9.32, *P* < 0.0005], on number of responses on the alcohol lever. Number of lever-responses during the last session of the extinction-responding phase was virtually identical in the four rat groups subsequently treated with 0, 5, 10, and 20 mg/kg KK-92A (Figure 5B). In the reinstatement session, presentation of the alcohol-associated stimulus complex reinstated lever-responding in the vehicle-treated rat group: the number of lever-responses averaged indeed 26.9 ± 4.2 and was approximately four times higher than that recorded in the same rat group during the last session of the extinction-responding phase (*P* < 0.0001) (Figure 5B). Acute treatment with KK-92A suppressed, in a dose-related manner, lever-responding in the reinstatement session; *post hoc* test indicated that statistical significance was reached by treatment with all three doses [5 (*P* < 0.0005), 10 (*P* < 0.0001), and 20 (*P* < 0.0001) mg/kg KK-92A]. The magnitude of the suppressing effect of 5, 10, and 20 mg/kg KK-92A on lever-responding averaged approximately 55, 80, and 85%, respectively (Figure 5B).

### Experiment 4: Testing KK-92A on Spontaneous Locomotor Activity

#### Time-Course Data

**FIGURE 6:**
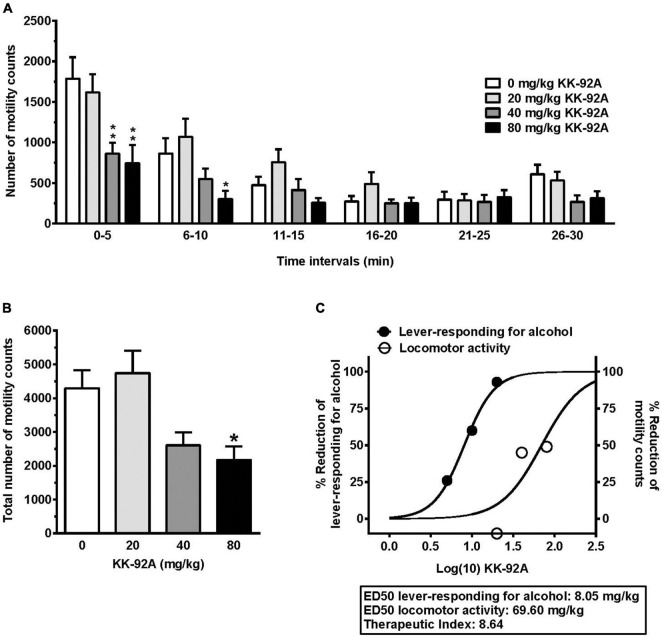
Effect of acute treatment with the positive allosteric modulator of the GABA_B_ receptor, KK-92A, on spontaneous locomotor activity in female Sardinian alcohol-preferring rats **(A,B)** and dose-response curves and calculated ED_5__0_s for KK-92A-induced hypomotility and reducing effect on alcohol self-administration **(C)**. Rats were initially trained to lever-respond for oral alcohol (15% v/v, in water) [Fixed Ratio (FR) 5 (FR5)] and water (FR1) in daily 30-min self-administration sessions. Once lever-responding had stabilized, rats were exposed to the locomotor-activity session. Specifically, rats were exposed to a computer-operated, photocell-equipped cage, to which they were unfamiliar. KK-92A was administered intraperitoneally 30 min before the start of the locomotor-activity session. The locomotor-activity session lasted 30 min. The measured variable was the total number of counts (photocell breaks) recorded automatically by the apparatus. In panel **(A)**, data are expressed as mean ± SEM of number of motility counts in six 5-min time intervals in *n* = 9–10 rats; *P < 0.05 and **P < 0.0001 in comparison to the rat group treated with 0 mg/kg KK-92A (Tukey’s test). In panel **(B)**, data are expressed as mean ± SEM of total number of motility counts over the entire locomotor-activity session in *n* = 9–10 rats; *P < 0.05 in comparison to the rat group treated with 0 mg/kg KK-92A (Tukey’s test). Data depicted in panel **(C)** are plotted as (i)% reduction in spontaneous locomotor activity [data from panel **(B)**] and (ii)% reduction in lever-responding for alcohol under the FR5 schedule of reinforcement (data from Figure 1A). EC_5__0_s were calculated by 4-parameter (top *plateau*, bottom *plateau*, middle or logEC_50_, and slope) logistic non-linear regression from sigmoidal dose-response curves using GraphPad 6 (GraphPad Software; La Jolla, CA, United States); bottom and top constraint equal to 0 and 100%, respectively, was used for curve fitting. Therapeutic index (TI) was calculated according to the following formula: “Hypomotility” ED_50_/“Reduction of lever-responding for alcohol” ED_50_.

Acute treatment with KK-92A reduced the number of motility counts in female sP rats [*F*_dose_(3, 35) = 5.81, *P* < 0.005; *F*_time_(5, 175) = 35.39, *P* < 0.0001; *F*_interaction_(15, 175) = 2.91, *P* < 0.0005] (Figure 6A). *Post hoc* test indicated that the reducing effect of KK-92A was limited to (i) the two highest doses tested (40 and 80 mg/kg) at the first time interval (0–5 min) and (ii) the dose of 80 mg/kg at the second time interval (6–10 min). Conversely, the number of motility counts was never affected by treatment with 20 mg/kg KK-92A (i.e., the highest dose tested in Experiments 1–3).

#### Cumulated Data and Calculation of the Therapeutic Index

Acute treatment with KK-92A reduced, in a dose-related manner, the total number of motility counts recorded over the 30-min session in female sP rats [*F*(3, 35) = 5.85, *P* < 0.005] (Figure 6B). *Post hoc* test indicated that statistical significance was reached only by treatment with 80 mg/kg KK-92A (*P* < 0.05), with a tendency toward a reduction after treatment with 40 mg/kg KK-92A. Conversely, the total number of motility counts recorded in the rat group treated with 20 mg/kg KK-92A was virtually identical to that recorded in vehicle-treated rats.

Data on KK-92A-induced hypomotility, together with those on KK-92A-induced suppression of alcohol self-administration under the FR5 schedule of reinforcement (Experiment 1A; Figure 1A), were used to establish a therapeutic index (TI) for KK-92A (Figure 6C). TI was calculated according to the following formula: “Hypomotility” ED_50_/”Reduction of lever-responding for alcohol” ED_50_ (for details on ED_50_ calculation, see the legend of Figure 6); accordingly, TI for KK-92A resulted to be equal to 8.64.

### Experiment 5: Testing KK-92A on Blood Alcohol Levels

**FIGURE 7:**
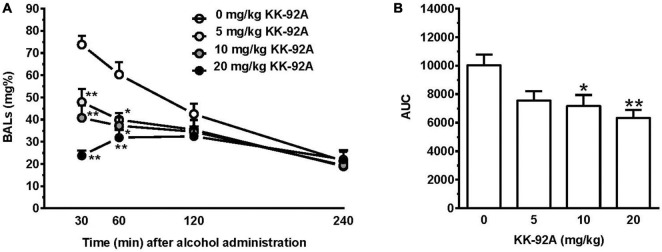
Effect of acute pretreatment with the positive allosteric modulator of the GABA_B_ receptor, KK-92A, on blood alcohol levels (BALs) in female Sardinian alcohol-preferring rats. Rats were initially trained to lever-respond for oral alcohol (15% v/v, in water) [Fixed Ratio (FR) 5 (FR5)] and water (FR1) in daily 30-min self-administration sessions. Once lever-responding had stabilized, KK-92A was administered intraperitoneally 30 min before the intragastric administration of 1 g/kg alcohol (15%, v/v). Blood samples were collected from the tip of the rat tail at 30, 60, 120, and 240 min after alcohol administration and analyzed by means of an enzymatic system. In panel **(A)**, BALs were expressed in mg%. Each point is the mean ± SEM of *n* = 10 rats. *P < 0.05 and **P < 0.01 in comparison to the rat group treated with 0 mg/kg KK-92A at the corresponding time (Tukey’s test). In panel **(B)**, data on the area under the curve of BAL time-course are expressed as (h*μg/ml). Each bar is the mean ± SEM of *n* = 10 rats. *P < 0.05 and **P < 0.005 in comparison to the rat group treated with 0 mg/kg KK-92A (Tukey’s test).

Acute pretreatment with KK-92A reduced, in a dose-related manner, BALs produced in female sP rats by acute, intragastric administration of 1 g/kg alcohol [*F*_dose_(3, 36) = 9.93, *P* < 0.0001; *F*_time_(2.20, 79, 93) = 45.34, *P* < 0.0001; *F*_interaction_(9, 108) = 7.48, *P* < 0.0001] (Figure 7A). *Post hoc* test indicated that the reducing effect of KK-92A on BALs was (i) limited to the first two recording times (30- and 60-min) and (ii) of larger magnitude (~70%) in the rat group treated with 20 mg/kg KK-92A at the 30-min recording time.

In close agreement with the above results, acute pretreatment with KK-92A also reduced the area under the curve of BAL time-course [*F*(3, 36) = 5.22, *P* < 0.005] (Figure 7B). *Post hoc* test indicated that statistical significance was reached by treatment with 10 (*P* < 0.05) and 20 (*P* < 0.005) mg/kg KK-92A. The magnitude of the reducing effect of 10 and 20 mg/kg KK-92A on the area under the curve of BAL time-course averaged approximately 30 and 40%, respectively.

## Discussion

In agreement with the working hypothesis of this study, data from Experiments 1A, 2, and 3 indicate that acute treatment with the GABA_B_ PAM, KK-92A, effectively reduced operant oral alcohol self-administration and cue-induced reinstatement of alcohol-seeking behavior in selectively bred alcohol-preferring sP rats. At the two highest doses (10 and 20 mg/kg) the reducing effect of KK-92A emerged as a virtually complete suppression of lever-responding for alcohol, amount of self-administered alcohol, breakpoint for alcohol, and reinstatement of alcohol seeking. In Experiments 1A and 2, latency to achieving the first alcohol reinforcer was considerably prolonged by treatment with 20 mg/kg KK-92A, suggesting that this dose of KK-92A suppressed the urge to seek for and consume alcohol. Analysis of cumulative response patterns from Experiments 1A (Figure 1D) and 2 (Figure 4D) provides additional insights on KK-92A action: in comparison to vehicle treatment, administration of all three doses of KK-92A resulted in (i) less steep curves (suggestive of a reduced frequency in lever-responding for alcohol), and (ii) lower *plateau* values (suggesting that fewer ratios were completed before lever-responding for alcohol ended). In Experiment 1A, the complete flatness of cumulative response pattern over the first 10 min of the session, observed after treatment with 20 mg/kg KK-92A, suggests that this dose of KK-92A abolished the typical “front-loading” of alcohol-drinking pattern of sP rats exposed to alcohol self-administration sessions under the FR schedule of reinforcement.

Acute treatment with KK-92A also decreased operant self-administration of a sucrose solution, the reinforcing properties of which were comparable to those of alcohol (number of lever-responses for alcohol and sucrose solution were indeed highly similar in vehicle-treated rats of Experiments 1A and 1B). KK-92A was however less potent and effective in reducing sucrose than alcohol self-administration: in the “sucrose” experiment, (i) reduction in lever-responding for sucrose solution was induced only by treatment with 20 mg/kg KK-92A and (ii) magnitude of the reducing effect of 20 mg/kg KK-92A on lever-responding for sucrose solution was limited to approximately 60% (compared to the approximately 95% suppression recorded in the “alcohol” experiment).

The limited selectivity of KK-92A effect on alcohol self-administration was somewhat unexpected for the following two main reasons. First, most of the GABA_B_ PAMs tested to date have been reported to reduce alcohol self-administration with no effect on self-administration of highly palatable sucrose, saccharin, sweetened-milk, or chocolate solutions (e.g., Filip et al., 2007; Maccioni et al.2007, 2008b, 2009, 2010, 2012, 2015, 2019b; Leite-Morris, 2013; see however Augier et al., 2017; Maccioni et al., 2017, 2019a). Second, treatment with the same doses of KK-92A tested in the present study resulted to be totally ineffective on operant self-administration of regular food pellets in rats (Li et al., 2017). Together, these data are suggestive of a peculiar ability of KK-92A to affect the reinforcing properties of highly palatable foods; this hypothesis is currently under experimental evaluation in our laboratories. These further analyses will also include investigation on whether treatment with KK-92A may alter palatability of sweet foods.

The suppressing effect of KK-92A on these alcohol- and sucrose-related behaviors was likely not influenced by any concurrent sedative or motor-incoordinating effect, which might have disrupted the regular rate of lever-responding. Data from Experiment 4 indicate indeed that hypolocomotion occurred at doses of KK-92A higher than those found to suppress alcohol and sucrose self-administration and reinstatement of alcohol seeking. More specifically, comparison of data from Experiments 1A and 4 resulted in a TI higher than 8, suggestive of a relatively large separation between the doses of KK-92A inducing the “desired” pharmacological effects (i.e., reduction of lever-responding for alcohol) and those inducing the “unwanted” adverse effects (i.e., sedation and reduced spontaneous locomotion).

Results of Experiments 1A, 2, and 3 extend to KK-92A a series of previous experimental data on the ability of the GABA_B_ PAMs, CGP7930, GS39783, BHF177, *rac*-BHFF, ADX71441, COR659, CMPPE, ORM-27669, and ASP8062, to decrease the reinforcing and motivational properties of alcohol and abolish cue-induced reinstatement of alcohol seeking in rats and mice (for references, see section “Introduction”). To our understanding, this extension should not be intended as just the mere generalization of previous data to a further GABA_B_ PAM; it is rather the demonstration that *all* GABA_B_ PAMs, most chemically unrelated to each other (see Mugnaini and Corelli, 2016; Nieto et al., 2021), produce highly similar effects on different alcohol-motivated behaviors in rodents, suggesting that reduction of alcohol seeking and drinking is a major feature of the pharmacological profile of the entire class of GABA_B_ PAMs. This conclusion, together with the notion that all these experimental data were collected using animal models with demonstrated predictive validity for specific aspects of human AUD, confer to GABA_B_ PAMs a promising therapeutic potential for AUD. Notably, ASP8062 has already been tested in two different Phase 1 clinical trials, proving to be safe, well-tolerated, and with good CNS penetration in healthy subjects (Walzer et al., 2020, 2021). ASP8062 is currently under investigation in a Phase 1 clinical trial to assess its potential interaction with alcohol in healthy subjects (ClinicalTrials.gov, 2019). ASP8062 might therefore be the first GABA_B_ PAM available to test whether the large and consistent body of preclinical evidence on the *anti*-alcohol effects of GABA_B_ PAMs translates to AUD patients.

The results of Experiment 1C indicate that treatment with a *per se* ineffective dose of KK-92A (2.5 mg/kg) potentiated the effect of baclofen (also given at a *per se* ineffective dose: 1 g/kg) on alcohol self-administration. Combination of KK-92A and baclofen produced indeed a 25–30% reduction, in comparison to all other treatment combinations, in number of responses on the alcohol lever and amount of self-administered alcohol. These results provide further confirmation that GABA_B_ PAMs augment *in vivo* the pharmacological activation of GABA_B_ binding site (see Urwyler, 2016; Nieto et al., 2021). They are also in agreement with two previous sets of data on the ability of the combination of (i) sub-threshold doses of CGP7930 (10 mg/kg, i.p.) and baclofen (2 mg/kg, i.p.) to reduce alcohol self-administration in selectively bred alcohol-preferring Indiana P rats (Liang et al., 2006), and (ii) *per se* ineffective doses of GS39783 (5 mg/kg, i.p.) or *rac*-BHFF (5 mg/kg, i.p.) and baclofen (1 mg/kg, i.p.) to reduce alcohol self-administration in sP rats (Maccioni et al., 2015). The results of these “combination” experiments (Liang et al., 2006; Maccioni et al., 2015; present study) apparently possess translational interest, as they suggest that treatment with low doses of a GABA_B_ PAM would potentiate the suppressing effect of baclofen on alcohol craving and consumption; this would permit to lower baclofen dose, maintaining its therapeutic effects unaltered while likely limiting its side-effects.

The results of the present study extend to alcohol previous data on the ability of KK-92A to ameliorate different nicotine-motivated behaviors in rats. More specifically, our US laboratory recently demonstrated that acute treatment with KK-92A (0, 5, 10, and 20 mg/kg; i.p.) decreased the number of nicotine infusions and breakpoint for nicotine in rats trained to self-administer nicotine intravenously under both FR and PR schedules of reinforcement (Li et al., 2017); acute treatment with KK-92A (0, 10, and 20 mg/kg; i.p.) also inhibited cue-induced reinstatement of nicotine seeking (Li et al., 2017). Notably, KK-92A effects were selective for nicotine, as no dose of KK-92A altered—even minimally—self-administration of and reinstatement of seeking behavior for regular food pellets (Li et al., 2017).

Inhibition of behaviors sustained by different drugs of abuse appears to be another remarkable, shared feature of the entire GABA_B_-PAM class. Indeed, and in addition to the above “nicotine” data on KK-92A (Li et al., 2017), it has been reported that treatment with CGP7930, GS39783, BHF177, *rac*-BHFF, CMPPE, and COR659 attenuated (i) operant intravenous self-administration of cocaine (Smith et al., 2004; Filip et al., 2007) and nicotine (Paterson et al., 2008; Vlachou et al., 2011), (ii) cocaine-primed and cue-induced reinstatement of cocaine seeking (Filip and Frankowska, 2007; Vengeliene et al., 2018), (iii) cue-induced reinstatement of nicotine seeking (Vlachou et al., 2011), (iv) context-driven seeking for cocaine (Halbout et al., 2011), (v) the lowering effect of cocaine (Slattery et al., 2005) and nicotine (Paterson et al., 2008) on threshold for intracranial self-stimulation, (vi) conditioned place preference induced by cocaine (de Miguel et al., 2019), amphetamine (Halbout et al., 2011), methamphetamine (Voigt et al., 2011), and nicotine (Mombereau et al., 2007), and (vii) locomotor activity stimulated by cocaine (Lhuillier et al., 2007; de Miguel et al., 2019; Lobina et al., 2021), amphetamine (Wierońska et al., 2011; Lobina et al., 2021), nicotine (Lobina et al., 2011, 2021), and morphine (Lobina et al., 2021) in rats and mice (for review, see Frankowska et al., 2016; Li and Slesinger, 2021).

In the majority of studies testing GABA_B_ PAMs on alcohol self-administration in rats and mice, and undeniably in all studies conducted in our Italian laboratory with sP rats, the magnitude of the decreasing effect of GABA_B_ PAMs on lever-responding for alcohol never exceeded 40–50%, featuring a reduction—rather than a suppression—of the reinforcing and motivational properties of alcohol (e.g., Maccioni et al., 2007, 2008b, 2009, 2019b). This relatively limited efficacy has been explained by the use-dependent mechanism of action of GABA_B_ PAMs: GABA_B_ PAMs potentiate endogenously released GABA, being ineffective in activating GABA_B_ receptors *per se* (see Urwyler, 2011, 2016). Therefore, their action depends on GABA concentration in the synaptic cleft, and the halving of a given *in vivo* effect, rather than its suppression, is likely the maximal behavioral consequence of GABA_B_ PAM-induced potentiation of extracellular GABA. Conversely, the effect of KK-92A on alcohol self-administration emerged as a marked suppression, as clearly depicted by the approximately 95% reduction in lever-responding for alcohol induced by treatment with 20 mg/kg KK-92A in Experiment 1A (Figure 1A). A possible explanation for this high efficacy may reside in the peculiar ago-allosteric profile of KK-92A. Recent *in vitro* assays demonstrated indeed that, beside potentiating GABA-induced cellular responses (GABA_B_-PAM activity), KK-92A also displayed distinct, intrinsic agonistic activity, activating the GABA_B_ receptor in the absence of GABA (Li et al., 2017). The suppressing effect of KK-92A on alcohol self-administration may therefore be the sum of two converging actions at the GABA_B_ receptor: (i) agonistic activity, resembling the suppressing effect of the prototypic GABA_B_ receptor agonist, baclofen, on alcohol-related behaviors (see Colombo and Gessa, 2018); (ii) positive allosteric modulation. The agonistic component of KK-92A might also be responsible for the reducing effect of KK-92A on sucrose self-administration, replicating the ability of baclofen to affect sucrose self-administration in rats at the same doses that reduced alcohol self-administration (e.g., Anstrom et al., 2003; Janak and Gill, 2003; Maccioni et al., 2005, 2008b; Echeverry-Alzate et al., 2021).

Reinstatement of alcohol seeking apparently deserves a separate mention. Indeed, the few studies conducted to date to test the effects of GABA_B_ PAMs on cue- and stress-induced reinstatement of alcohol seeking have reported that treatment with ADX71441 (Augier et al., 2017), CMPPE (Vengeliene et al., 2018; Maccioni et al., 2019b), and COR659 (Maccioni et al., 2019a) completely suppressed, rather than merely reducing, lever-responding in the reinstatement session. The suppressing effect of KK-92A on cue-induced reinstatement of alcohol seeking, observed in Experiment 3, is entirely consistent with these literature data. Together, these results may be interpreted to suggest that reinstatement of alcohol seeking is highly sensitive to positive allosteric modulation of the GABA_B_ receptor, theoretically highlighting GABA_B_ PAMs as a drug of choice for treating craving for alcohol, loss of control over alcohol, and relapse episodes into heavy drinking. These data also suggest the relevant role of GABA_B_ receptor in the neural substrate mediating the reinstatement of alcohol seeking behavior, as previously suggested by the suppressing effect of baclofen on cue-induced reinstatement of alcohol seeking in rats (Maccioni et al., 2008a; Vengeliene et al., 2018).

Data from Experiment 5 indicate that pretreatment with all three doses of KK-92A reduced BALs generated by the acute intragastric administration of 1 g/kg alcohol. This effect was evident over the first hour after alcohol administration (corresponding to 90 min after KK-92A injection), while it vanished at the subsequent recording times, likely paralleling the progressive reduction of KK-92A plasma levels and efficacy. To our knowledge, only two previous studies investigated the effect of GABA_B_ PAMs on alcohol metabolism: (i) acute, intragastric administration of *rac*-BHFF suppressed BALs produced in sP rats by the acute intragastric administration of 1 g/kg alcohol (Maccioni et al., 2010); (ii) neither acute nor repeated intraperitoneal injection of GS39783 altered BALs produced in DBA/2J mice by acute or repeated administration of 2 g/kg alcohol (Kruse et al., 2012). Among the several methodological differences of these three studies (Maccioni et al., 2010; Kruse et al., 2012; present study), the route of alcohol administration might offer a key to explain the observed discrepancies. Since the two studies reporting a reduction in BALs used the intragastric route of alcohol administration, it is reasonable to hypothesize that positive allosteric modulation of GABA_B_ receptors located in the gastrointestinal tract (Nakajima et al., 1996; Castelli et al., 1999) interfered with gastric emptying and/or intestinal motility, possibly altering alcohol absorption and metabolism.

The suppressing effect of KK-92A on alcohol self-administration (Experiment 1A) is somewhat difficult to reconcile with its effect on BALs (Experiment 5). Treatment with a drug reducing BALs is indeed expected to result in an increase, rather than a decrease, in alcohol seeking and drinking, as rats should increase their lever-responding for alcohol and amount of self-administered alcohol to possibly achieve the usual brain concentrations of alcohol and perceive the subsequent psychopharmacological effects. KK-92A-induced suppression of alcohol self-administration under the FR schedule of reinforcement and reduction of BALs appear to be opposite effects, with the former overtaking the latter: the central effects of KK-92A on the reinforcing and motivational properties of alcohol impacted the rat behavior to a greater extent than its peripheral effects on alcohol absorption and metabolism. Conversely, there was no apparent relationship between the central and peripheral effects of KK-92A in the results of Experiments 2 and 3, in which lever-responding resulted in modest and pharmacologically irrelevant intake (PR schedule of reinforcement) or even absence (reinstatement of alcohol seeking) of self-administered alcohol, ruling out that KK-92A action on alcohol absorption and metabolism could have influenced the rat behavior.

The experiments conducted in the present study used ovariectomized female sP rats. The choice of (small) female, instead of (heavy) male, rats was dictated by several practical advantages, described in detail elsewhere (Lorrai et al., 2019); here we mention solely the aptness of commercially available operant chambers, usually too narrow to accommodate animals as large as adult male sP rats. Ovariectomy was performed to avoid any possible influence of ovarian hormones on the several alcohol- and sucrose-related behaviors investigated in this study as well as on alcohol metabolism. While this has surely been an advantageous simplification of the experimental design of this first investigation, additional studies are now needed to assess and compare KK-92A effects in male and intact (non-ovariectomized) female sP rats. The results of these studies will be of relevance also in terms of the possible translatability of these findings to AUD patients.

The few studies to date that have investigated the neural substrates mediating the suppressing effects of GABA_B_ PAMs on alcohol-related behaviors suggested a role for the mesolimbic dopamine “reward” system. More specifically, it has been proposed that activation of GABA_B_ receptors located in the ventral tegmental area (VTA) likely hyperpolarizes the mesolimbic dopamine neurons, thus preventing their alcohol-induced stimulation and dopamine release in the nucleus accumbens, and decreasing the rewarding and reinforcing properties of alcohol (see Phillips and Reed, 2014; Colombo and Gessa, 2018; Maccioni and Colombo, 2019). This conclusion is supported by data demonstrating that intra-VTA microinjection of CGP7930, GS39783, and BHF177 effectively decreased alcohol self-administration (Maccioni et al., 2018), alcohol seeking (Leite-Morris et al., 2009; Leite-Morris, 2013), and accumbal dopamine release stimulated by cues predictive of alcohol availability (Leite-Morris, 2013) in rats. It is reasonable to hypothesize that this mechanism also applies to the suppressing effects of KK-92A on alcohol-motivated behaviors observed in the present study. An additional, possible mechanism of action is based on the recent observation that alcohol-dependent rats had reduced amygdalar levels of the GABA transporter GAT3 and, subsequently, high concentrations of extracellular GABA (Augier et al., 2018). It has been proposed that activation of amygdalar presynaptic GABA_B_ receptors by baclofen—and GABA_B_ PAMs, we add—would inhibit GABA release, reducing extracellular GABA levels, restoring the enhanced tonic inhibition of amygdala and, in the end, decreasing alcohol drinking (Spanagel, 2018; Marti-Prats et al., 2021).

In conclusion, the results of the present study demonstrate that treatment with non-sedative doses of the novel, selective GABA_B_ PAM, KK-92A, potently and effectively suppressed operant oral alcohol self-administration and cue-induced reinstatement of alcohol seeking in alcohol-preferring sP rats. Treatment with KK-92A also potentiated the reducing effect of baclofen on alcohol self-administration. These data extend to KK-92A a large and entirely consistent body of experimental evidence on the ability of GABA_B_ PAMs to decrease several alcohol-motivated behaviors in rodents, strengthening the notion that amelioration of alcohol-motivated behaviors is a major feature of the entire class of GABA_B_ PAMs. Additionally, these data extend to alcohol previous experimental data on the ability of KK-92A to decrease nicotine self-administration and cue-induced reinstatement of nicotine seeking in rats (Li et al., 2017), widening the *anti*-addictive profile of KK-92A.

## Data Availability Statement

All raw data of this article will be made available on request by the corresponding authors, without undue reservation.

## Ethics Statement

The experimental procedures employed in the present study fully complied with European Directive No. 2010/63/EU and subsequent Italian Legislative Decree No. 26, March 4, 2014, on the “Protection of animals used for scientific purposes.”

## Author Contributions

GC, PMa, and PMc conceived the study. GC and PMa designed the experimental approach. KK, HL, and SY synthesized and performed the compound analysis of KK-92A. PMa and JB performed the *in vivo* experiments. PMa analyzed the *in vivo* data. GC, GG, and PMc wrote the manuscript. All authors contributed to the article and approved the submitted version.

## Conflict of Interest

The authors declare that the research was conducted in the absence of any commercial or financial relationships that could be construed as a potential conflict of interest.

## Publisher’s Note

All claims expressed in this article are solely those of the authors and do not necessarily represent those of their affiliated organizations, or those of the publisher, the editors and the reviewers. Any product that may be evaluated in this article, or claim that may be made by its manufacturer, is not guaranteed or endorsed by the publisher.

## Supplementary Material

Supplementary Figure 1 Structure and compound analysis of KK-92A. KK-92A was synthesized in gram-scale with >99% purity (as determined by HPLC) according to the procedure described in detail by Li et al. (2017). The chemical analysis (^1^H and ^13^C NMR, HPLC-MS) of in-house synthesized KK-92A matched the reported data.
