# Supplementary material for: The Novel Positive Allosteric Modulator of the GABAB Receptor, KK-92A, Suppresses Alcohol Self-Administration and Cue-Induced Reinstatement of Alcohol Seeking in Rats
Source: Front Cell Dev Biol. 2021 Oct 28;9:727576. doi: 10.3389/fcell.2021.727576 (PMC8585307; doi:10.3389/fcell.2021.727576)

Supplementary Material

Supplementary Figure 1. Compound Analysis

^{13}C NMR of KK-92A

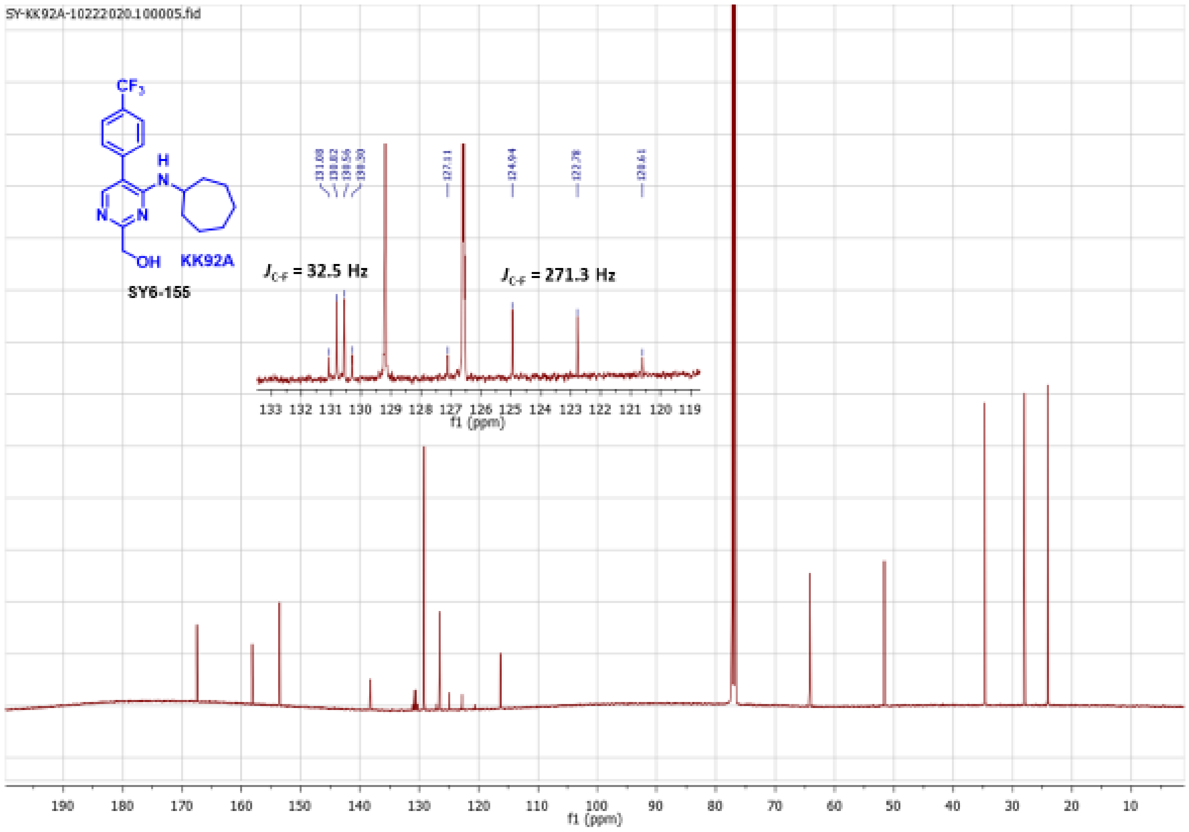

^{1}H NMR of KK-92A

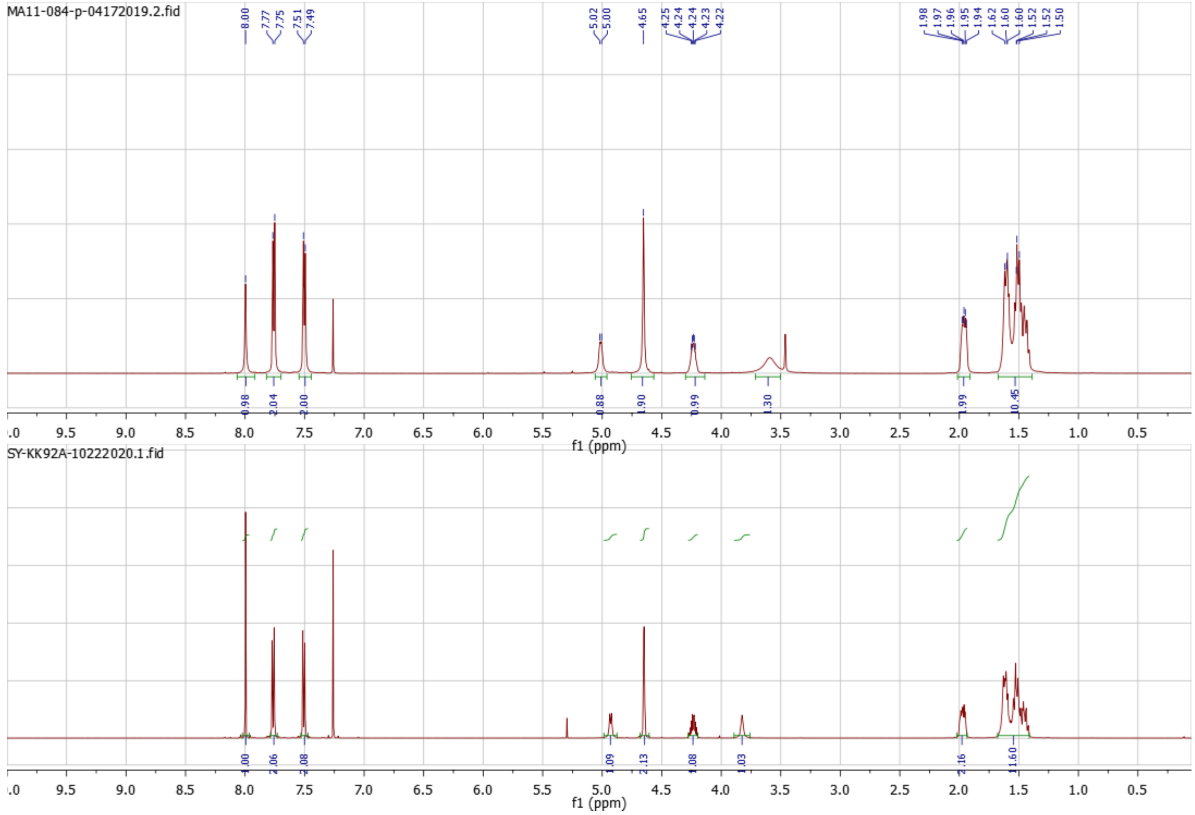

HPLC analysis of KK-92A: Purity of KK-92A > 99%. Method: CH3OH 60% H2O 40%, TFA 0.1%, 1ml 60 min.

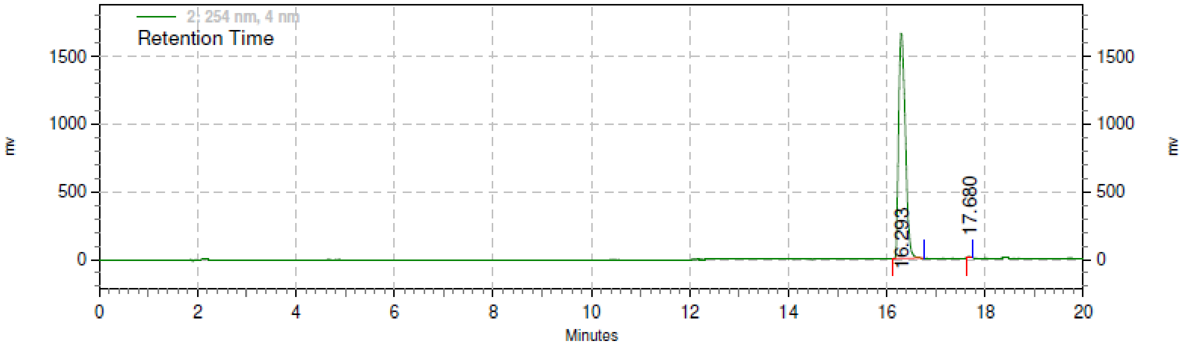

2: 254 nm, 4 nm
Results

Retention Time	Area	Area %	Height	Height %
16.293	15908930	99.80	1665367	99.55
17.680	32241	0.20	7523	0.45
Totals				
	15941171	100.00	1672890	100.00

HPLC-MS of KK-92A

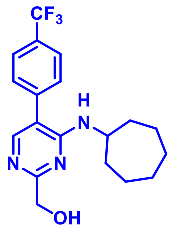

KK-92A

Exact Mass: 365.1715

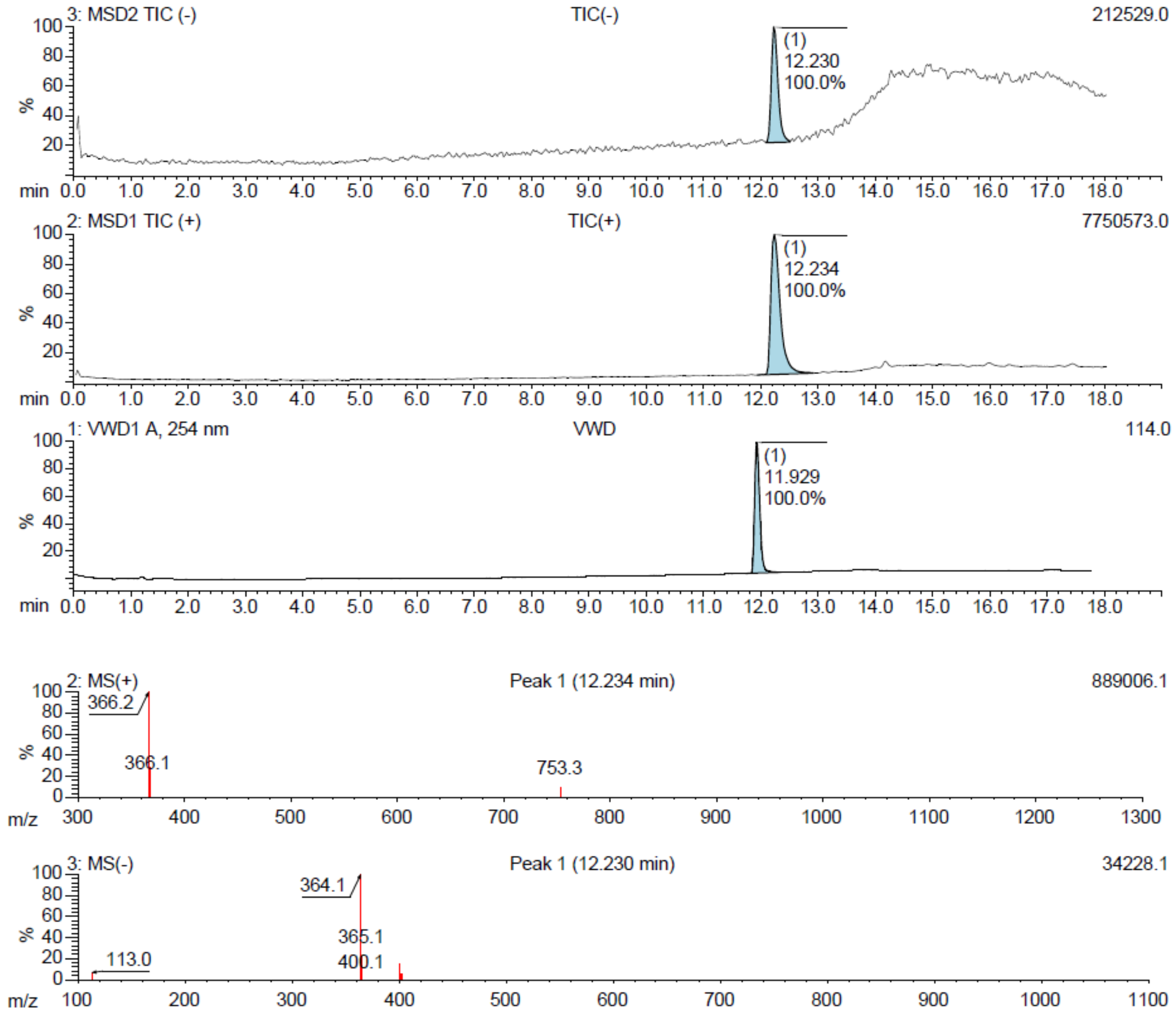

Supplement: Supplementary Figure 1 — Structure and compound analysis of KK-92A. KK-92A was synthesized in gram-scale with >99% purity (as determined by HPLC) according to the procedure described in detail by Li et al. (2017). The chemical analysis (1H and 13C NMR, HPLC-MS) of in-house synthesized KK-92A matched the reported data. [file Data_Sheet_1.PDF]
